# Hypertension Management in Patients with Chronic Kidney Disease

**DOI:** 10.14797/mdcvj.1119

**Published:** 2022-09-06

**Authors:** Sean A. Hebert, Hassan N. Ibrahim

**Affiliations:** 1 Department of Surgery, Division of Immunology and Organ Transplantation, The University of Texas Health Science Center at Houston (UTHealth), Houston, Texas, US

**Keywords:** hypertension, chronic kidney disease, masked hypertension, ambulatory blood pressure monitor

## Abstract

Hypertension and chronic kidney disease are closely linked. Patients with chronic kidney disease have hypertension almost universally and uncontrolled hypertension accelerates the decline in kidney function. The pathophysiology of hypertension in chronic kidney disease is complex, but is largely related to reduced nephron mass, sympathetic nervous system overactivation, involvement of the renin-angiotensin-aldosterone system, and generalized endothelial dysfunction. Consensus guidelines for blood pressure targets have adopted a blood pressure <120/80 mm Hg in native chronic kidney disease and <130/80 mm Hg in kidney transplant recipients. Guidelines also strongly advocate for renin-angiotensin-aldosterone system blockade as the first-line therapy.

## Introduction

Hypertension is a common finding in individuals with chronic kidney disease (CKD), afflicting 65% to 85% of patients and increasing as kidney function declines.^1^ The causes of CKD, including salt retention, overt hypervolemia, sympathetic overactivity, and endothelial dysfunction, contribute to this high prevalence. Moreover, uncontrolled hypertension has a graded relationship with cardiovascular disease and remains a leading cause of cardiac morbidity and mortality. In this article, we review the classification of blood pressure (BP), goals of BP reduction, and hypertension management in those with CKD.

## BP Classification

An office blood pressure (OBP) of 140/90 mm Hg is thought to correlate with a 24-hour average ambulatory blood pressure measurement (ABPM) of 130/80 mm Hg and a mean home BP of 135/85 mm Hg.^2^ With the change in hypertension diagnosis thresholds in the 2017 American Heart Association/American College of Cardiology (AHA/ACC) guidelines, an OBP < 120/80 mm Hg is still considered normal, but an OBP of 120–129/ <80 mm Hg is elevated, and an OBP ≥ 130/80 mm Hg is consistent with hypertension.^2^ An OBP reading of 130/80 mm Hg is thought to correlate with a 24-hour average reading of 125/75 mm Hg, and the recommended 24-hour ABPM targets are: 24-hour mean BP ≤ 125/75 mm Hg, a daytime BP ≤ 130/80 mm Hg, and a nighttime BP ≤ 110/65 mm Hg with appropriate nocturnal dipping.^2^ When both OBP and ABPM are available, individuals may be classified into one of four groups: controlled (normal office and ABPM), white coat hypertension (elevated office and normal ABPM), masked hypertension (normal office and elevated ABPM), and sustained hypertension (elevated office and ABPM).

Masked hypertension (30–60%) in CKD has a higher prevalence compared to the general population (10–25%).^3^ In the Chronic Renal Insufficiency Cohort (CRIC) study, 1,502 participants with CKD had ABPM profiles available for review. Masked hypertension was associated with more cardiovascular events and a more rapid decline in kidney function after a mean follow-up of 6.72 years.^4^ Increasing use of ABPM or home blood pressure monitoring (HBPM) in clinical CKD practice will help confirm treatment responses and perhaps better characterize risk for future target organ damage.

## Proper BP Measurement Technique

### “Standardized” Office Blood Pressure Measurement

Routine in-office BP measurements served as the traditional method for hypertension diagnosis and treatment decisions until recent recommendations favored implementation of standardized practices.^2,5^ Standardized OBP measurements should include: (1) appropriate cuff size (bladder should encircle 80% of the bare arm); (2) proper positioning (patient’s arm should be supported at the level of the heart while seated with back supported, legs uncrossed, and both feet flat on floor); (3) patient preparation (patient should relax for 5 minutes, abstain from caffeine, exercise, and smoking in the preceding 30 minutes, and ensure his or her bladder is empty; and (4) multiple measurements (≥ 2 readings at least 1–2 minutes apart) in both arms while using a validated device that is appropriately calibrated.^2,5^ Importantly, these measurements should be done without the white coat provider being in the room.

Many studies have demonstrated the lack of correlation between routine and standardized OBP measurements.^6,7,8^ Agarwal et al. compared routine versus standardized BP measurements in 275 participants from the Systolic Blood Pressure Intervention Trial (SPRINT). The mean systolic blood pressure (SBP) and diastolic blood pressures (DBP) in routine office measurements were 12.7 and 12 mm Hg higher, respectively, than the standardized measurements performed on the same day.^8^ Moreover, studies have implicated poor proficiency by medical staff in following standardized BP measurement protocols, therefore re-training should occur on a regular basis.^9,10,11^

### Ambulatory BP Monitoring

Out-of-office BP measurements should complement standardized OBP measurements and include both ABPM and HBPM. Briefly, ABPM involves the application of an appropriately sized BP cuff to the nondominant arm with BP measurements taken every 20 to 30 minutes for a 24- to 48-hour period. While following their normal daily routine, individuals are instructed to keep their arm still during measurements and to keep a diary of sleep and wake periods.^12^ ABPM provides a more robust estimate of the 24-hour, daytime and nighttime readings, including BP variables such as dipping and BP decline > 10% during sleep. Both nocturnal hypertension and non-dipping status are prevalent in CKD and are associated with higher risk of cardiovascular morbidity and mortality.^13^

## Goals of BP Reduction and Consensus Targets

**Figure 1:**
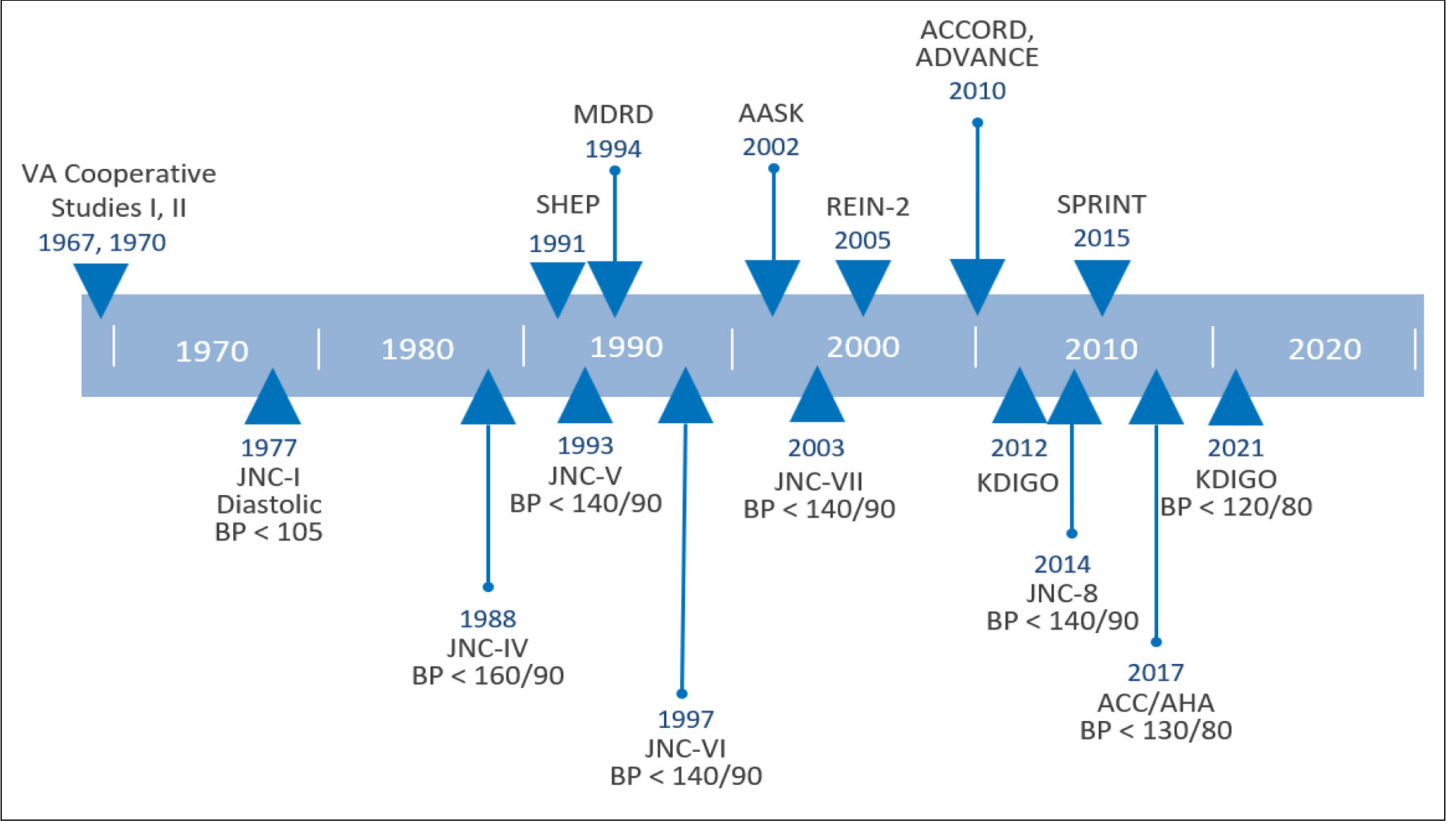
United States blood pressure guidelines for chronic kidney disease (CKD) hypertension targets over time. Above timeline: Hypertension trials with CKD participants from 1960 to 2018 (not all inclusive). Below timeline: Guidelines and hypertension treatment targets from 1960 to 2021. CKD: chronic kidney disease; AASK: African American Study of Kidney Disease and Hypertension; ACCORD: Action to Control Cardiovascular Risk in Diabetes; ADVANCE: The Action in Diabetes and Vascular Disease: Preterax and Diamicron Controlled Evaluation trial; HTN: hypertension; KDIGO: Kidney Disease Improving Global Outcomes; MDRD: Modification of Diet in Renal Disease; REIN-2: Blood-Pressure Control for Renoprotection in Patients with Non-diabetic Chronic Renal Disease; SPRINT: Systolic Blood Pressure Intervention Trial; SHEP: Systolic Hypertension in the Elderly Program; VA: Veterans Affairs. Adapted from Chang A et al. Blood Pressure Goals in Patients with CKD. CJASN Jan 2019, 14 (1) 161–169.

The optimal BP targets for CKD hypertension treatment have evolved as new research accumulates (Figure 1).^14^ In September 2015, the randomized SPRINT trial stopped before completion—after the interim analysis showed the group assigned to an intensive systolic BP goal < 120 mm Hg had a 25% lower cardiovascular disease risk and 27% lower all-cause mortality compared with the standard group assigned to systolic BP < 140 mm Hg.^15^ Subsequent guidelines from the AHA/ACC in 2017 recommended a BP goal of <130/80 mm Hg in patients with CKD and those with increased cardiovascular risk. The AHA/ACC chose 130 mm Hg instead of BP < 120 mm Hg due to concerns that routine clinic visit BP measurements are unlikely to be measured in a standardized approach such as that used in the SPRINT trial.^16^ In addition, there were more hypotensive and acute kidney injury events requiring emergency department visits among participants in the intensive group. Citing these concerns, the 2018 European Society of Hypertension and European Society of Cardiology (ESH/ESC) guidelines recommended a goal of SBP between 130 and 139 mm Hg in CKD hypertension.^17^

Newer recommendations from the 2021 Kidney Disease Improving Global Outcomes (KDIGO) clinical practice guidelines have favored more intensive treatment to a SBP of < 120 mm Hg.^5^ This recommendation followed subgroup analysis that tested the two BP targets (SBP < 120 vs SBP < 140 mm Hg) among 2,600 CKD patients in SPRINT and showed lower composite cardiovascular outcome (HR 0.81; 95% CI, 0.63-1.05) and lower mortality (HR 0.72; 95% CI, 0.53-0.99) after median follow-up of 3.3 years in the intensive arm.^18^ Fortunately, kidney outcomes such as the development of end-stage kidney disease (ESKD) or a greater than 50% reduction in estimated glomerular filtration rate (eGFR) were not more pronounced between the two groups, with one exception: a more pronounced eGFR decline in the intensive group in the first 6 months (-0.47 vs -0.32 mL/min/1.73m^2^/year; *P* < .03), attributed to an acute hemodynamically mediated decline in the renal blood flow, without further appreciable change over the remainder of the study.^18^

The SPRINT trial differed from three previous trials comparing BP targets in CKD due to heterogeneity in primary outcomes. The Modification of Diet in Renal Disease (MDRD) trial, the African American Study of Kidney Disease and Hypertension (AASK) trial, and the Blood-Pressure Control for Renoprotection in Patients with Non-diabetic Chronic Renal Disease (REIN-2) trial used kidney outcomes (ESKD and reduced eGFR) as primary outcomes instead of cardiovascular outcomes.^19,20,21^ These studies had few cardiovascular and mortality events, making data synthesis challenging for evidence-based practice guidelines. A meta-analysis performed by Malhotra et al. later examined mortality among CKD subgroups, including these three trials, and showed a lower mortality in the intensive BP control group (HR 0.86; 95% CI, 0.76-0.97; *P* = .01).^22^ However, intensive BP targets among all four studies showed no benefit for reducing kidney outcomes.^23^ Taken together, an intensive BP target seems appropriate for reductions in cardiovascular complications, particularly given the higher cardiovascular risk in people with CKD.

The 24-hour ABPM can help ensure BP targets are met by identifying two specific BP phenotypes in hypertensive CKD patients: masked uncontrolled hypertension (MUCH) and white coat hypertension (WCH). MUCH, defined as normal clinic blood pressures but uncontrolled out-of-office blood pressures, is grossly underrecognized. Up to 60% of clinically normotensive CKD patients are uncontrolled out of the office and possibly face increased cardiovascular (CV) risk and accelerated kidney function decline.^24,25^ Individuals with WCH have uncontrolled clinic blood pressures but normal out-of-office blood pressures and represent up to 30% of clinically uncontrolled hypertensive CKD patients. The diagnostic failure of these two phenotypes have led to under- and overtreatment, respectively.^12^ Ku et al. showed the clinical relevance of these conditions among 610 participants in the AASK trial, which revealed a U-shaped association between clinic and ambulatory SBP difference and an increased mortality but no ESKD risk.^26^ However, the generalizability of this trial to all hypertensive CKD patients is unknown. The ongoing 4-year prospective randomized MASked-unconTrolled hypERtension management based on office BP or on ambulatory blood pressure measurement (MASTER) study may help clarify this ambiguity if it indicates that ABPM-based treatment strategies slow target organ damage and reduce future CV events.^27^

## Treatment

### Non-pharmacologic

Dietary interventions and daily exercise are adjuncts to pharmacologic therapies and should be the first step to hypertension management. The Dietary Approaches to Stop Hypertension (DASH) diet, which favors fruits and vegetables over saturated fats, has led to modest BP declines in hypertensive individuals.^28^ Reducing sodium intake to less than 2 grams daily may lower SBP by 5 to 10 mm Hg, and increasing potassium intake to more than 3 grams daily may be additive in those who are salt sensitive.^28,29^ However, dietary sodium reduction should not be a universal recommendation as it will have little impact on BP in those with CKD and salt-losing nephropathies.^30^ More importantly, high potassium content of many of these BP-friendly foods may even provoke hyperkalemia.

Regular exercise should be encouraged to help lower blood pressure and improve CV health in CKD, not to mention its beneficial effects on quality of life. Prior studies suggest that exercise implementation may improve eGFR at 12 months or slow CKD progression.^31,32^ This effect on eGFR, however, was inconsistent in newer studies.^33,34,35,36^ To align guidelines, KDIGO and AHA/ACC now recommend “a total duration of 150 minutes of moderate intensity physical activity (resistance or aerobic) per week.”^5,37^ Individuals with CKD that have limited exercise ability due to their comorbidities should perhaps have this exercise target modified accordingly. Weight loss of more than 5 kg can enhance the favorable exercise benefits by lowering the SBP by 5 mm Hg.^38^ For those with obstructive sleep apnea, a recent meta-analysis showed nocturnal continuous positive airway pressure treatment can have a modest reduction in SBP by up to 5 mm Hg in the most severe cases.^39^ This is highly relevant as sleep disturbances and sleep apnea are extremely common in CKD.^40^

### Pharmacologic

Antihypertensive medications are almost always needed in patients with CKD. When initiating antihypertensive medication, one should consider starting two drugs from different classes, particularly in those with Stage 2 hypertension (≥ 140/90 mm Hg).^2^ Little to no evidence is available on outcomes comparing different drug combinations in CKD. The typical primary agents are renin-angiotensin-system inhibitors (RAAS), which include angiotensin-converting enzyme inhibitors (ACEi), and angiotensin receptor blockers (ARB), as well as calcium channel blockers (CCB) and diuretics. Beta blockers, on the other hand, lack evidence of benefit, increase risk of new-onset type 2 diabetes, and perhaps should be avoided as first-line therapy unless indicated for cardiovascular disease.^41,42^

ACEi and ARBs are the mainstays of CKD hypertension management, particularly in those with albuminuria (urine albumin > 300 mg/d).^43^ Ruggenenti et al. showed ramipril’s salutary effect in slowing eGFR decline among 166 proteinuric patients (> 3 g/d) compared with placebo (0.51 ± 0.09 vs 0.76 ± 0.10 mL/min/1.73 m^2^ per month, *P* < .03) in the Gruppo Italiano di Studi Epidemiologici in Nefologia (GISEN) study, or Ramipril Efficacy In Nephropathy (REIN) follow-up study.^44^ A meta-analysis of 119 randomized controlled trials showed ACEi and ARBs reduced kidney failure by 39% and 30%, and reduced major cardiovascular events by 18% and 24%, respectively, versus placebo.^45^ The combination of ACEi and ARBs is discouraged because it is associated with higher rates of acute kidney injury (AKI) and hyperkalemia.^46^ In the ESKD population, dosing adjustment should be considered in those without residual kidney function. For example, lisinopril dosing should be three times weekly instead of daily, preferably after hemodialysis due to potential for being removed with dialysis.^47^

There is less consensus concerning the optimal second-line antihypertensives. Dihydropyridine calcium channel blockers, such as amlodipine, are commonly used as an adjunct to an ACEi or ARB on the basis of their synergistic ability to reduce BP.^48^ There is also evidence to suggest nondihyropyridine CCBs (eg, verapamil and diltiazem) have a more pronounced antiproteinuric effect and may be a reasonable therapeutic option for proteinuric CKD despite maximal doses of ACEi or ARBs.^49^ CCBs such as nifedipine should be avoided particularly in those with proteinuria as they may transmit systemic pressures to the glomerular space more readily.

Diuretics are also a reasonable second-line therapy choice, especially in those with reduced kidney function and hypervolemia. The prevailing dogma has been that thiazide diuretics lose effectiveness at a lower GFR, and guidelines have recommended changing from a thiazide to a loop diuretic at GFR values below 30 mL/min/1.73 m^2^.^50^ However, the evidence against thiazide use in advanced CKD is weak. A recent randomized control trial showed that, for patients with stage 4 CKD, the addition of chlorthalidone to traditional antihypertensives reduced SBP by 11 mm Hg (95% CI, -13.9 to -8.1) at 12 weeks.^51^ Chlorthalidone and indapamide are preferred over hydrochlorothiazide due to their longer half-lives and higher potency, and reductions in GFR may be met with increased drug dosages.^52^

Resistant hypertension, defined as uncontrolled BP despite using three antihypertensive medications, one of which is a diuretic, is common with CKD.^53^ Prior to diagnosing an individual with resistant hypertension, clinicians should confirm accurate clinic BP measurements as well as use out-of-office BP measurements to exclude pseudo-resistance. Mineralocorticoid receptor antagonists (MRA) such as spironolactone and eplerenone have been found to reduce BP in resistant hypertension.^54^ However, hyperkalemia may be a concern, particularly when added to a background of ACEi or ARBs in the setting of reduced GFR, as seen with advanced CKD. AMBER trial results showed that use of the oral potassium binder patiromer enabled more patients with resistant hypertension and CKD to continue spironolactone.^55^ In addition, the recent FIDELIO trial showed that treatment with the nonsteroidal MRA finerenone had lower risks of CKD progression and lower CV events in those with type 2 diabetes and CKD.^56^

Among patients with CV disease and CKD, there may be indications for drugs such as beta-blockers, though dual alpha and beta blockade may be superior in BP reduction due to its additional vasodilatory effect.^57^ Sodium-glucose cotransporter 2 inhibitors also have been associated with significant reductions in home BP in individuals with type 2 diabetes mellitus, as well as patients with resistant hypertension, and have shown a laudable reduction in kidney disease progression and CV mortality.^58,59^

Diurnal variation of BP higher in the early morning, followed by a decrease in BP later in the evening to night, particularly during sleep, is well described.^60^ The recommendation for nocturnal administration of antihypertensives has been increasing recently due to its hypothesized impact on reducing CV risk by lowering morning BP surges. A meta-analysis by Wang et al. showed nocturnal administration of BP medications resulted in a significant decrease in nocturnal SBP by 3.2 mm Hg (95% CI; -5.41 to -0.94) and a significant reduction in nocturnal DBP by 1.4 mm Hg (95% CI; -2.05 to -0.69). However, it showed no difference with regard to CV risk and all-cause mortality.^61^ The Hygia Chronotherapy trial was perhaps the largest study to examine this issue among more than 19,000 European patients who were hypertensive. The initial results showed a 45% reduction in CV risk despite only marginal BP reductions.^62^ Unfortunately, issues with their study protocol and overall conclusions came to light and the authors have since retracted the article.^63^ There is also evidence that nocturnal BP lowering can be hazardous with increased risk of myocardial and cerebral infarcts, particularly in elderly populations.^64^ Given the lack of CKD patients in these studies and uncertainty over benefit versus harm, no firm recommendations can be made regarding nighttime ingestion of antihypertensive agents.^5^

### BP Management in Kidney Transplant Recipients

Different from the non-transplant CKD hypertension guidelines, the most recent 2021 KDIGO CKD hypertension guidelines state that a BP target < 130/80 mm Hg remains a reasonable goal for kidney transplant recipients.^5^ This stems from a lack of studies targeting different BP goals in the kidney transplant population and concerns over eGFR decline, AKI, and incident CKD among those in the intensive BP arm of the SPRINT trial. Kidney transplant recipients with a solitary, denervated kidney purportedly could be placed at increased risk of such events with intensive BP lowering, though this has not been substantiated by clinical data.

Similar to the non-transplant CKD population, HBPM and ABPM should be used to complement OBP measurements for hypertension management. Recommended first-line antihypertensives include ARBs and CCBs. Dihydropyridine CCBs (eg, amlodipine) are typically favored in the immediate perioperative period until kidney function stabilizes. Importantly, non-dihydropyridines (eg, verapamil, diltiazem) might interfere with the metabolism of immunosuppressant medications, particularly the calcineurin inhibitors cyclosporine and tacrolimus, as well as the mammalian target of rapamycin inhibitors sirolimus and everolimus, and careful monitoring of their drug levels is required.^65^

Meta-analyses have shown dihydropyridine CCB and ARB use were associated with a 38% and 65% reduction in graft loss, respectively, over a mean follow-up of 25 months, while non-dihydropyridines had no effect.^5^ The mechanism for ARBs and reduced graft loss is not well understood. Ibrahim et al. showed losartan use was associated with less interstitial expansion, a precursor to graft fibrosis, though this was not statistically significant.^66^ Importantly, ARB use was well tolerated in kidney transplant recipients with minimal hyperkalemia.

## Conclusion

Hypertension management in CKD lowers incident CV risk and reduces kidney disease progression. Existing guidelines have moved closer to consensus BP targets and place more emphasis on accurate BP measurements and more dependence on home and ABPM. Pharmacologic therapies offer varying degrees of risk reduction in CKD, and lifestyle interventions should be encouraged to augment these health benefits. Most importantly, patient engagement with out-of-office BP measurements, as well as more informed and shared decision making, will lead to long-term successes.

## Key Points

- The goal blood pressure for hypertension management in chronic kidney disease is < 120/80 mm Hg to reduce cardiovascular disease risk.
- Masked uncontrolled hypertension is highly prevalent in CKD patients, and out-of-office measurements such as home or ambulatory blood pressure monitoring are needed for diagnosis.
- Angiotensin-converting enzyme inhibitors and angiotensin receptor blockers are first-line for hypertension treatment in proteinuric CKD.
- Thiazide and thiazide-like diuretics have a role in hypertension management even in advanced kidney disease.
